# Supplementary figures and images for: Ocular and uteroplacental pathology in a macaque pregnancy with congenital Zika virus infection
Source: PLoS One. 2018 Jan 30;13(1):e0190617. doi: 10.1371/journal.pone.0190617 (PMC5790226; doi:10.1371/journal.pone.0190617)

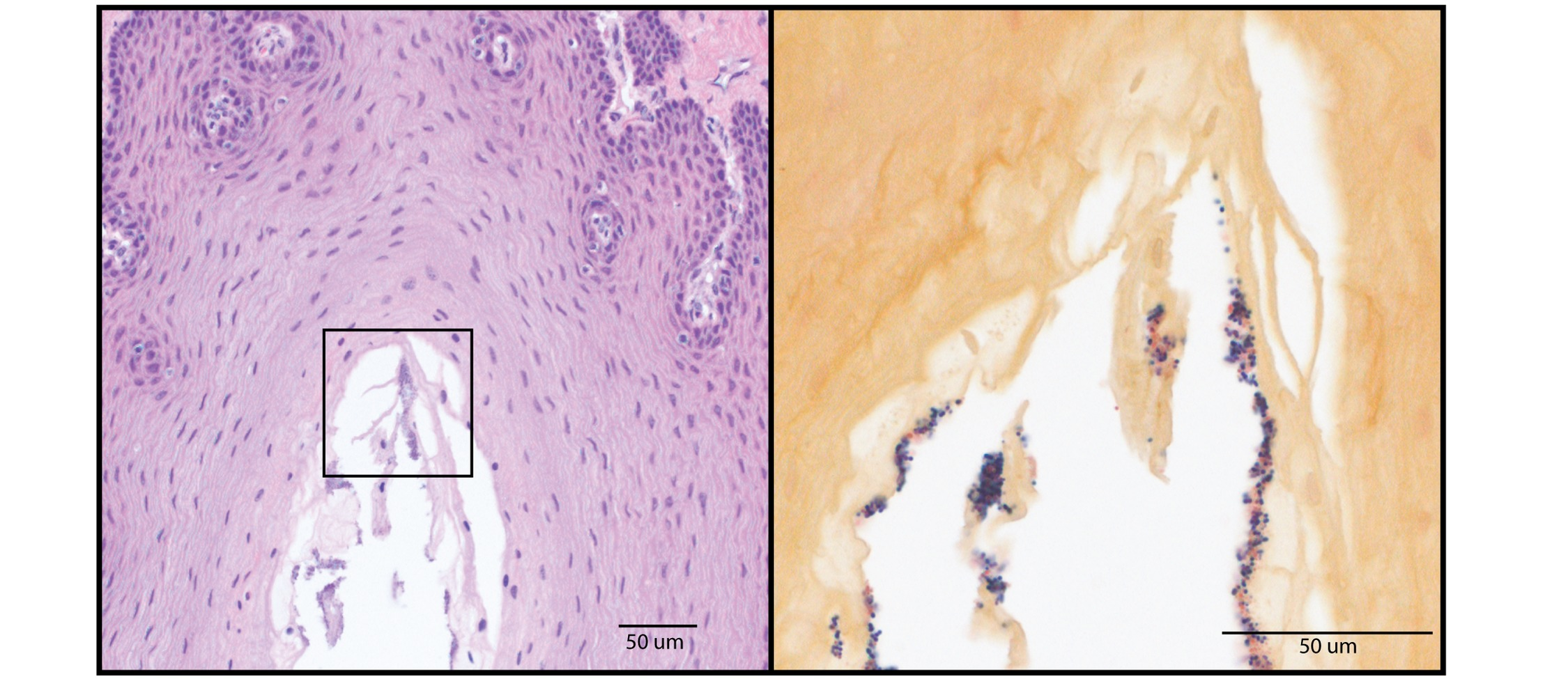

Supplement: S1 Fig — A) H&E image of the fetal esophagus. B) Gram stain of a section of the fetal esophagus epithelial surface, where gram positive cocci were abundant. (TIF) [file pone.0190617.s001.tif]

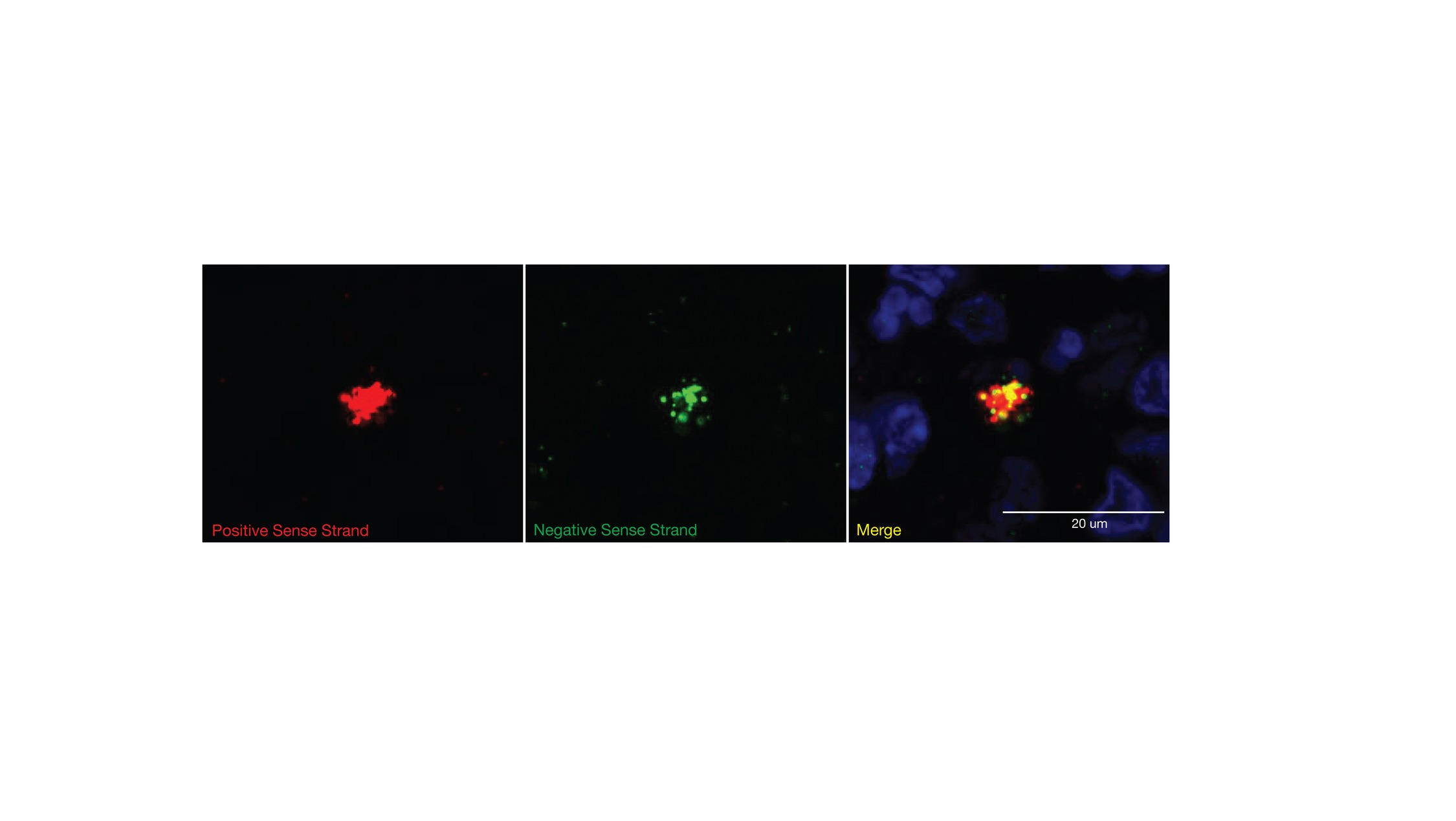

Supplement: S2 Fig — (TIF) [file pone.0190617.s002.tif]

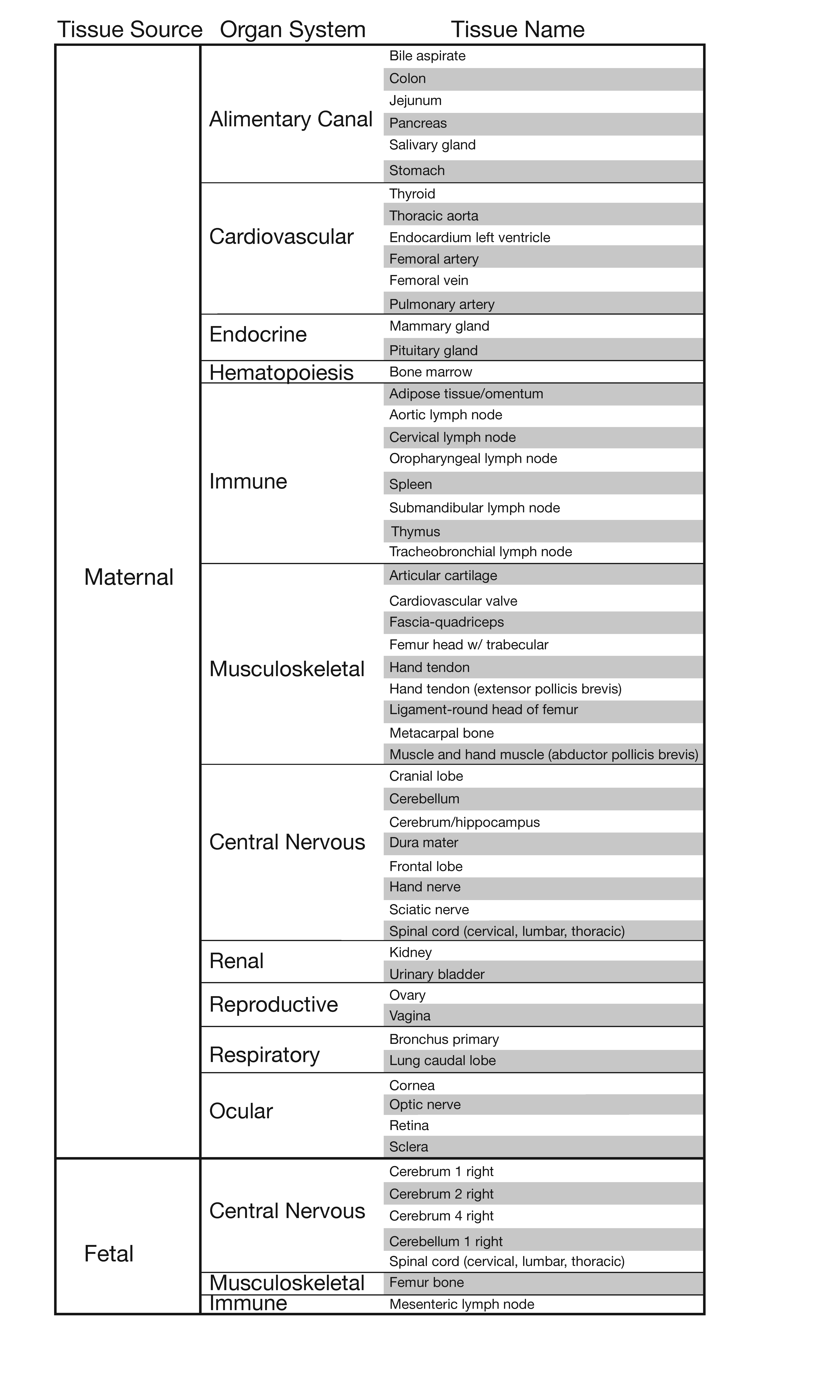

Supplement: S1 Table — (TIF) [file pone.0190617.s003.tif]

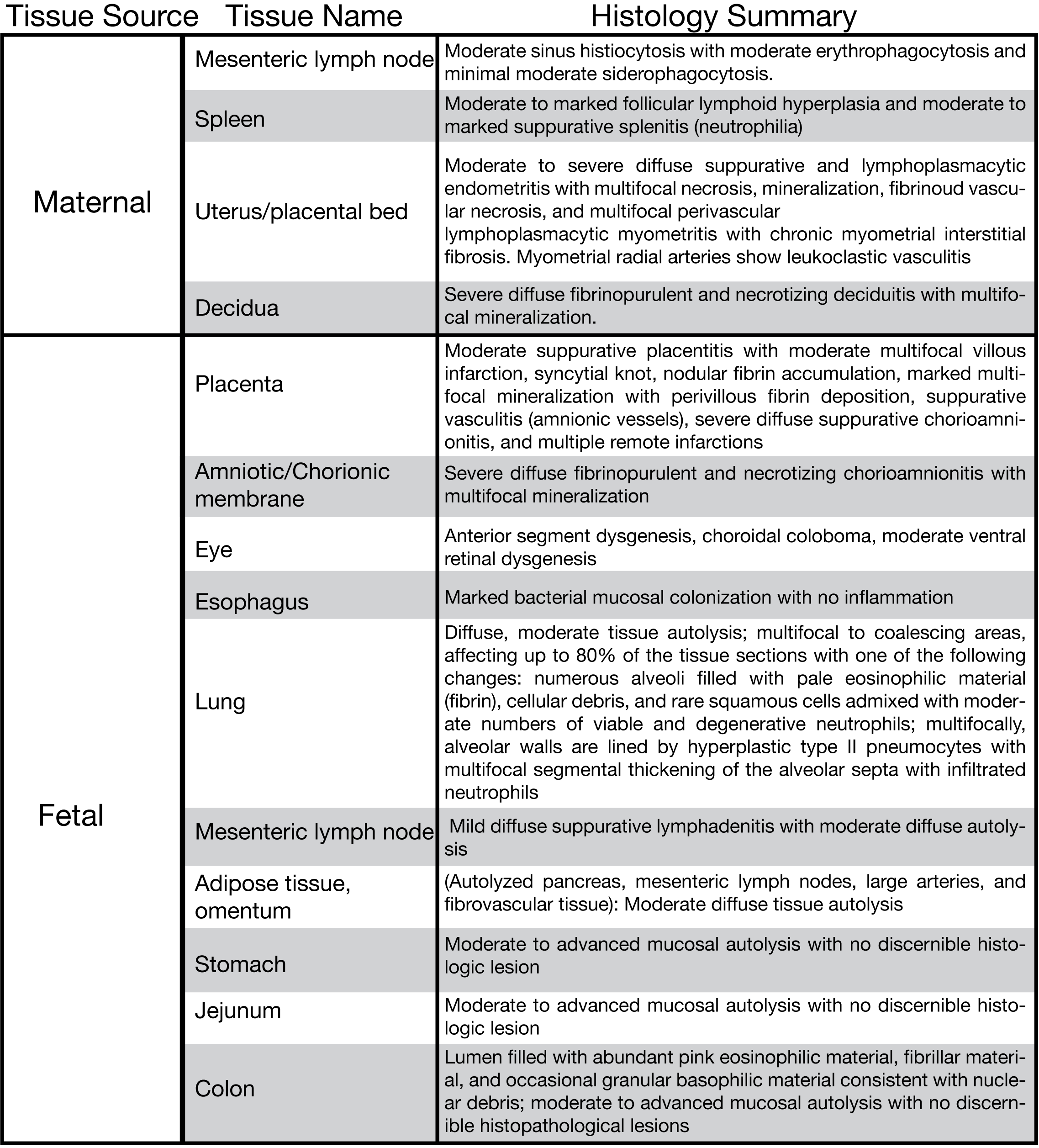

Supplement: S2 Table — (TIF) [file pone.0190617.s004.tif]
